# Transcutaneous vagus nerve stimulation modulates depression‐like phenotype induced by high‐fat diet via P2X7R/NLRP3/IL‐1β in the prefrontal cortex

**DOI:** 10.1111/cns.14755

**Published:** 2024-05-16

**Authors:** Shaoyuan Li, Yuzhengheng Zhang, Yu Wang, Zixuan Zhang, Chen Xin, Yifei Wang, Peijing Rong

**Affiliations:** ^1^ Institute of Acupuncture and Moxibustion, China Academy of Chinese Medical Sciences Beijing China; ^2^ Institute of Basic Research in Clinical Medicine, China Academy of Chinese Medical Sciences Beijing China

**Keywords:** depression‐like phenotype, high‐fat diet, microglia, P2X7R/NLRP3/IL‐1β signaling pathway, transcutaneous auricular vagus nerve stimulation

## Abstract

**Background:**

Depression is a common psychiatric disorder in diabetic patients. Depressive mood associated with obesity/metabolic disorders is related to the inflammatory response caused by long‐term consumption of high‐fat diets, but its molecular mechanism is unclear. In this study, we investigated whether the antidepressant effect of transcutaneous auricular vagus nerve stimulation (taVNS) in high‐fat diet rats works through the P2X7R/NLRP3/IL‐1β pathway.

**Methods:**

We first used 16S rRNA gene sequencing analysis and LC–MS metabolomics assays in Zucker diabetic fatty (ZDF) rats with long‐term high‐fat diet (Purina #5008) induced significant depression‐like behaviors. Next, the forced swimming test (FST) and open field test (OFT) were measured to evaluate the antidepressive effect of taVNS. Immunofluorescence and western blotting (WB) were used to measure the microglia state and the expression of P2X7R, NLRP3, and IL‐1β in PFC.

**Results:**

Purina#5008 diet induced significant depression‐like behaviors in ZDF rats and was closely related to purine and inflammatory metabolites. Consecutive taVNS increased plasma insulin concentration, reduced glycated hemoglobin and glucagon content in ZDF rats, significantly improved the depressive‐like phenotype in ZDF rats through reducing the microglia activity, and increased the expression of P2X7R, NLRP3, and IL‐1β in the prefrontal cortex (PFC).

**Conclusion:**

The P2X7R/NLRP3/IL‐1β signaling pathway may play an important role in the antidepressant‐like behavior of taVNS, which provides a promising mechanism for taVNS clinical treatment of diabetes combined with depression.

## Graphical abstract

Transcutaneous auricular vagus nerve stimulation can reduce the depression‐like behaviors of high‐fat diet‐induced ZDF rats by modulating the anti‐inflammatory effects generated by the P2X7R/NLRP3/IL‐1β signaling pathway in the PFC, which provides a promising mechanism for taVNS clinical treatment of diabetes combined with depression.

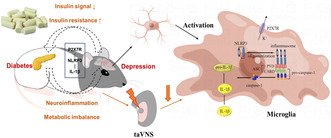

## 1 INTRODUCTION

Depressive mood disorder is one of the most common, highly prevalent, debilitating disorders, frequently comorbid with many chronic diseases and more often with metabolic disorders.^1^ Social defeat stress is a paradigm that has been widely used to induce a depressive phenotype and potentiate systemic insulin resistance in mice with diet‐induced obesity.^2^ Meanwhile, depression‐like behavior has been reported in a mouse model of obesity/metabolic disorders induced by long‐term high‐fat diet consumption.^3^ Although combination therapy with hypoglycemic agents and antidepressants^4^ is available, the risk of weight gain, pathoglycemia, and other adverse reactions limits its application.^5^ In 2005, invasive vagus nerve stimulation (VNS) has been approved by the US Food and Drug Administration to treat refractory depression.^6^ It has been shown that the subdiaphragmatic VNS can regulate depressive‐like behavior through inflammatory responses and neurotransmitter release, and it was observed that subdiaphragmatic vagotomy (SDV) blocked the onset of depressive‐like behavior in mice.^7^ However, VNS and SDV have been limited by postoperative complications.^8^ At the same time, the vagus nerve also plays an important role in controlling pancreatic hormone secretion by affecting glucagon and insulin secretion to regulate blood glucose.^9^ Transcutaneous auricular vagus nerve stimulation (taVNS) has been greatly verified for the treatment of depression^10^ and metabolic disturbance.^6^, ^11^, ^12^, ^13^

In recent years, the concept of the “gut‐brain axis” has emerged to summarize the bidirectional regulation between the gastrointestinal tract and the central nervous system.^14^ It integrates multiple modes of information transmission, such as neurotransmission, immune signaling, and inflammation. Research has shown that depressive‐like behavior in rats is associated with an increase in the abundance of pro‐depressant microbial species such as thick‐walled bacteria and a decrease in the abundance of antidepressant microbial species such as bifidobacteria, and a correlation with indicators of diabetes has been found for flora such as bacteroides phylum.^15^ This evidence suggests that changes in the amount of gut flora are associated with depression and metabolic diseases.

The association between depression and metabolic disorders has been extensively researched, with a deeper understanding of the underlying mechanisms. Increasing evidence indicates that insulin resistance, triggered by high‐fat diet (HFD) exposure, also manifests in the brain, affecting crucial physiological processes essential for mood regulation. Mice with a brain‐specific knockout of the insulin receptor (NIRKO mice) exhibited insulin resistance in the brain and displayed depressive‐like behaviors.^16^ Further research confirmed that central insulin resistance occurs in dopaminergic^17^ and serotonergic neurons.^18^ Inflammation has also been suggested to mediate the phenotypic overlap of metabolic disorders and depressive syndrome.^19^ Our previous studies have shown that taVNS can significantly inhibit hypothalamic P2Y1R expression and attenuate weight gain without decreasing food intake in Zucker diabetic fatty (ZDF) rats, which demonstrated that taVNS could be utilized to improve the function of the purinergic system.^20^

The P2X7 receptor plays an important role in the innate immune response by regulating the expression of inflammatory factors in the IL‐1 family. P2X7 receptors activate the NLRP3 inflammasome, and excessive activation of the NLRP3 inflammasome causes IL‐1β release, which in turn induces depressive‐like behaviors and insulin resistance in rats.^21^ The P2X7R/NLRP3/IL‐1β pathway is an important pathological mechanism of diabetes and depression.^22^ Microglia are the only immunocompetent cells with an immune function in the central nervous system (CNS). It is helpful for synaptogenesis and brain homeostasis, and can also be excessively activated in many conditions, such as inflammation, infection, trauma, and apoptosis. Inflammatory cytokine secretion from activated microglia into the extracellular environment involves neuroinflammation and mood disorder pathogenesis.^23^ The prefrontal cortex (PFC) is a crucial brain region for comorbid depression and metabolic disorders.^24^ Moreover, our previous studies showed that the antidepressive effect of taVNS might be related to the increased functional connectivity between the rostral anterior cingulate cortex and mPFC.^25^ However, the potential molecular mechanism of taVNS in the comorbid microglia‐driven P2X7R/NLRP3/IL‐1β signaling pathway in the PFC has been not completely elucidated. Therefore, in this study, we aimed to show that taVNS modulates the depression‐like phenotype induced by a high‐fat diet in ZDF rats and investigate the effect of taVNS on neuroinflammatory markers such as P2X7R, NLRP3, and IL‐1β in microglia to elucidate the central antidepressant mechanism of taVNS in ZDF rats.

## 2 METHODS

### 2.1 Ethics statement

All protocols for conducting animal experiments were approved by the Ethics Committee of the China Academy of Chinese Medical Sciences (NO. 20160616), and all procedures complied with the *National Institutes of Health Guide for the Care and Use of Laboratory Animals*.

### 2.2 Animals

Six‐week‐old ZDF rats and Zucker lean (ZL) rats were obtained from the Beijing Vital River Laboratory Animal Technology Co., Ltd. [License No. SCXK (Beijing) 2016‐0006]. All rats were housed at the Institute of Acupuncture and Moxibustion, China Academy of Chinese Medical Sciences in a controlled environment of 24 ± 2°C with a humidity of 55% ± 2%, and in quiet states maintained under a 12/12‐h light/dark cycle with ad libitum access to food and water (except when indicated). In consideration of the estrogen intervention, only male rats were chosen for this study.

### 2.3 Experimental protocol and groups

#### 2.3.1 Part I

**FIGURE 1:**
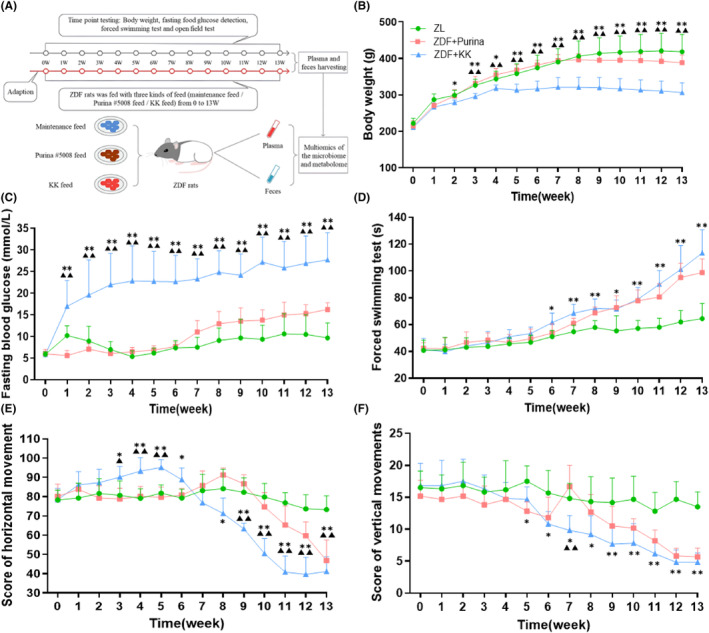
Comparison of depressive‐like behaviors in different diet groups. (A) Experimental procedure. ZDF, Zucker diabetes rat. (B and C) Different diets influence the change in body weight and the level of fasting blood glucose in ZDF rats. (D–F) Different diets influence depressive‐like behaviors in different groups. The values are presented as the mean ± SD. *One‐way ANOVA* with *Tukey's* post–hoc *test* was performed for statistical analysis. *N* = 6 rats for each group. *p < 0.05, **p < 0.01 vs. ZL group, ^▲▲^ *p* < 0.01 vs ZDF + Purina group. Statistical analysis of *one‐way ANOVA*: (B) 0 w, *p* = 0.4134; 1 w, *p* = 0.0697; 2 w, *p* = 0.0371; 3 w, *p* = 0.0017; 4 w, *p* = 0.0045; 5 w, *p* = 0.0006; 6 w, *p* = 0.0006; 7 w, *p* = 0.0004; 8 w, *p* = 0.0003; 9 w, *p* = 0.0002; 10 w, *p* = 0.0002; 11 w, *p* = 0.0002; 12 w, *p* = 0.0002; 13 w, *p* = 0.0001. (C) 0 w, *p* = 0.9432; 1 w, *p* = 0.0004; 2 w, *p* = 0.0014; 3 w, *p* < 0.0001; 4 w, *p* < 0.0001; 5 w, *p* < 0.0001; 6 w, *p* < 0.0001;7 w, *p* < 0.0001; 8 w, *p* < 0.0001; 9 w, *p* < 0.0001; 10 w, *p* < 0.0001; 11 w, *p* = 0.0002; 12 w, *p* < 0.0001; 13 w, *p* < 0.0001. (D) 0 w, *p* = 0.9137; 1 w, *p* = 0.8889; 2 w, *p* = 0.6117; 3 w, *p* = 0.4426; 4 w, *p* = 0.2123; 5 w, *p* = 0.1904; 6 w, *p* = 0.0296; 7 w, *p* = 0.0015; 8 w, *p* = 0.0014; 9 w, *p* = 0.0078; 10 w, *p* = 0.0078; 11 w, *p* < 0.0001; 12 w, *p* = 0.0002; 13 w, *p* < 0.0001. (E) 0 w, *p* = 0.8283; 1 w, *p* = 0.1872; 2 w, *p* = 0.1595; 3 w, *p* = 0.0149; 4 w, *p* = 0.0010; 5 w, *p* < 0.0001; 6 w, *p* = 0.0079; 7 w, *p* = 0.2134; 8 w, *p* = 0.0015; 9 w, *p* < 0.0001; 10 w, *p* < 0.0001; 11 w, *p* < 0.0001; 12 w, *p* < 0.0001; 13 w, *p* < 0.0001. (F) 0 w, *p* = 0.5853; 1 w, *p* = 0.3982; 2 w, *p* = 0.4163; 3 w, *p* = 0.2177; 4 w, *p* = 0.6605; 5 w, *p* = 0.0037; 6 w, *p* = 0.0297; 7 w, *p* = 0.0015; 8 w, *p* = 0.0401; 9 w, *p* = 0.0118; 10 w, *p* = 0.0029; 11 w, *p* = 0.0004; 12 w, *p* < 0.0001; 13 w, *p* < 0.0001.

Twenty ZDF rats were randomly divided into the ZDF + Purina group (32% fat, 13% protein, 55% carbohydrate; *N* = 10) and ZDF + KK group (17.9% fat, 17.5% protein, 48.5% carbohydrate; *N* = 10) compared with the ZL group (general maintenance diet #1022, *N* = 10). Behavioral tests, forced swimming test (FST) and open field test (OFT), were, respectively, measured every 2 weeks to evaluate the depressive‐like phenotype.^3^, ^26^, ^27^, ^28^, ^29^ Gut microbiota composition and metabolome characteristics were measured at the end of this experiment (Figure 1A).

#### 2.3.2 Part II

**FIGURE 2:**
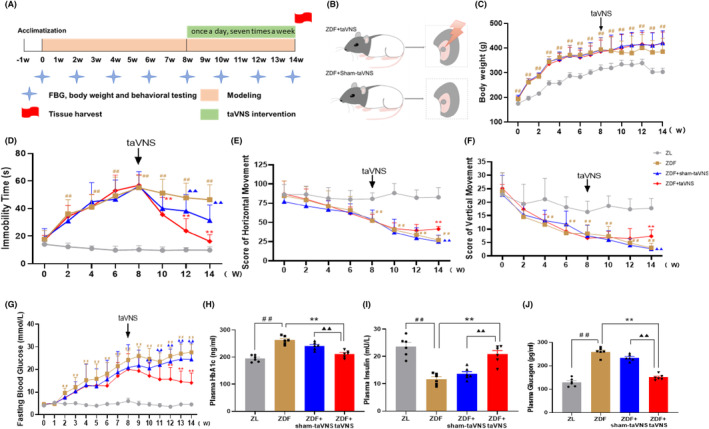
The effect of taVNS on the behavioral experiments and blood glucose‐related indexes of rats at different time points. (A) Experimental procedure. ZDF, Zucker diabetes rat. FBG, fasting blood glucose. taVNS, transcutaneous auricular vagus nerve stimulation. From 0 w to 14 w, FBG, OFT, and FST were measured every 2 weeks. taVNS or sham‐taVNS intervention was carried out every day during 8 w and 14 w; (B) Intervention of taVNS and sham‐taVNS. The skin receptive area of taVNS is in the auricular concha with auricular branch of vagus nerve distribution; (C) Effect of taVNS on the body weight at different time points; (D) Comparison of immobility time of rats at different time points; (E, F) Comparison of horizontal movement and vertical movement scores of rats in open field test at different time points; (G) Effect of taVNS on the fasting blood glucose at different time points; (H–J). Effect of taVNS on the concentration of plasma HbA1c, insulin, and glucagon. The values are presented as the mean ± SD. *One‐way ANOVA* with *Tukey's* post hoc *test* was performed for statistical analysis. *N* = 6 rats for each group. ^##^ *p* < 0.01 vs. ZL group, **p < 0.01 vs. ZDF group, ^▲▲^ *p* < 0.01 vs. ZDF + taVNS group.

To further confirm the mechanism of taVNS in diabetics with depression. Another 30 ZDF rats fed with high‐fat Purina #5008 diet were randomly divided into the ZDF group (ZDF), taVNS group (ZDF + taVNS), and sham‐taVNS group (ZDF + sham‐taVNS), with 10 rats per group, after acclimatization feeding for 1 week, compared with ZL group (Figure 2A). FST and OFT were measured to evaluate the antidepressive effect of taVNS. Immunofluorescence and western blotting (WB) were used to measure the microglia state and the expression of P2X7R, NLRP3, and IL‐1β in PFC.

### 2.4 Behavioral tests

The FST apparatus consisted of a cylindrical Plexiglas tank (60 cm × 30 cm × 45 cm). Each rat was placed in the tank (water temperature was 24–26°C), and the camera equipment was fixed outside the tank to record each rat's activity and the time of immobility for 3 min. Immobility is when a rat no longer struggles or just floats on the water's surface.^30^, ^35^, ^36^ The square arena of OFT was made of a 100 cm × 100 cm × 40 cm plastic board without special smell, characterized by a black wall and a black base, and the base was divided into equal squares of 20 cm × 20 cm by white stripes. A horizontal movement score and a vertical movement score were recorded for 3 min. An alcohol spray bottle and paper towels were used to clean up the odor and excrement at the end of each measurement.^31^, ^32^

### 2.5 Fecal sample collection and 16S rRNA gene sequencing analysis

The feces defecated were immediately collected and held in individual sterile Eppendorf tubes, quickly frozen in liquid nitrogen, and then stored at −80°C until DNA extraction and analysis. Microbial DNA was extracted from feces using Qubit dsDNA Assay kit. The purity and concentration of total DNA were tested by NanoDrop2000, followed by PCR amplification of the selected amplified region, the V3–V4 region of 16S rRNA, with the following primer sequences: 343F: TACGGRAGGCAGCAG; 798R: AGGGTATCTAATCCT. TACGGRAGGCAGCAG; 798R: AGGGTATCTAATCCT. The amplified products were detected by 2% agarose gel electrophoresis, followed by purification of the PCR products using the AxyPrep DNA Gel Extraction Kit and detection with a Quantus™ Fluorometer. Quantification was followed by Miseq library construction and sequencing. Quality control screening was performed on original downstream data to obtain high‐quality sequences, splicing was performed according to the overleap relationship between PE reads, operational taxonomic unit (OTU) clustering was performed on duplicate sequences according to 97% similarity principle to obtain OTU representative sequences, and further analysis of sequences was performed, and the above analysis was based on the Oebiotech cloud platform. Graphical plotting and statistical analyses were also performed on the Oebiotech cloud platform, and the differences were statistically significant at *p* < 0.05.

### 2.6 Plasma sample preparation and liquid chromatograph mass spectrometer

The samples stored at −80°C were thawed at room temperature, 100 μL of plasma was pipetted, 10 μL of each internal standard (L‐2‐chlorophenyl alanine, 0.3 mg/mL; Lyso PC 17:0, 0.01 mg/mL, both in methanol) was added, vortexed, and shaken for 10 s, and then 300 μL of protein precipitant methanol–acetonitrile (V:V = 2:1) was added, vortexed, and shaken for 1 min. After extraction, the extract was sonicated in an ice‐water bath for 10 min and left to stand at −20°C for 30 min, followed by centrifugation at 16,320 *g* for 10 min; 300 μL of supernatant was evaporated, then 400 μL of methanol–water (V:V = 1:4) was redissolved, vortexed for 30 s, sonicated for 2 min, and centrifuged again (16,320*g*, 4°C), and 150 μL supernatant was aspirated and filtered using a 0.22 μm organic phase pinhole filter and transferred to an LC injection vial and stored at −80°C until LC–MS analysis was performed. All samples were scanned by primary mass spectrometry and secondary mass spectrometry. Primary mass spectrometry was performed by the full scan with a resolution of 70,000 and a scan range of 100–1000 m/z; secondary mass spectrometry was performed by a data‐dependent secondary scan with a resolution of 17,500, and the cleavage mode was high‐energy collision‐induced dissociation with collision energies: 10, 20, and 40. The raw data from the mass spectrometry downcomers were imported into Progenesis QI v2.3 software (Nonlinear Dynamics, Newcastle, UK) for baseline filtering, peak identification, integration, retention time correction, peak alignment, and normalization. The data matrices of the mass‐to‐charge ratio, retention time, grouping information, and normalized peak area were obtained. The data matrices were imported into SIMCA–P 14.1 software for statistical analysis, PCoA, and orthogonal PLS–DA. Differential biomarkers were screened according to variable important projection values greater than 1.5 and a *P* value less than 0.05. For the identification of the discrepant compounds, Progenesis QI v2.3 software was used to obtain secondary fragmentation spectra fitting the molecular formula as well as this mass‐to‐charge ratio, search the Human Metabolome, Lipid maps, and Metlin databases, and compare the secondary spectra in databases based on the secondary mass spectra ion. The structure of the compound was determined based on the fragmentation information and the bond‐breaking pattern. The pathway enrichment analysis of the differential metabolites was performed by MetaboAnalyst 5.0. The metabolic pathways involved in differential metabolites were analyzed in combination with KEGG database and related literature.

### 2.7 TaVNS intervention

Rats in the taVNS group were exposed to continuous transcutaneous electrical stimulation once a day for 6 consecutive weeks using an electric apparatus (HANS‐200A, Nanjing Jisheng Medical Technology Co., Ltd.).

The stimulation parameters were: intensity 2 mA, frequency 2/15 Hz, sparse and dense waves, 30 min/session. The rats were subjected to electrical stimulation interventions under isoflurane inhalation anesthesia (4% concentration of isoflurane combined with 99.5% oxygen, the rats were induced to complete short‐acting general anesthesia by perfusion and subsequently moved to the sub‐channel, and anesthesia concentration was reduced to 2% for maintenance) (Hebei Nine Sent Pharmaceutical Co., Ltd., Hebei, China) from 14:00 to 16:00 each afternoon. Positive and negative self‐absorbing conductive electromagnets were connected and immobilized bilaterally in the auricular nail cavities of the rats in a noninvasive manner, and the observation of slight fluttering of the external auricle demonstrated the triumphant arrival of the stimulus.^33^ For the sham‐taVNS group, the stimulation sites were the same as the taVNS group but without electricity (Figure 2B).

### 2.8 Fasting blood glucose

Fasting blood glucose (FBG) was tested by tail‐tip blood sampling using a blood glucose meter (model: Contour TS), and the model was considered successful when FBG was ≧11.1 mmol/L.

### 2.9 Enzyme‐linked immunosorbent assay (ELISA)

Concentrations of plasma hemoglobinA1c (HbA1c), insulin, and glucagon were analyzed at week 14 after taVNS/sham‐taVNS intervention by ELISA (HbA1c, Cat. #EIA‐3394; insulin, Cat. #EIA‐3619; glucagon, Cat. #EIA‐3733).

### 2.10 WB

The segments were homogenized in an SDS sample buffer containing a mixture of proteinase inhibitors (Sigma). Protein samples were separated on SDS–PAGE gels and transferred to polyvinylidene difluoride filters. The filters were blocked with 3% milk and incubated overnight at 4°C with primary antibodies against of P2X7R (1:500, rabbit polyclonal, Allomone labs, APR‐004), NLRP3 (1:500, rabbit monoclonal, Abcam, ab214185), and IL‐1β (1:500, rabbit polyclonal, Abcam, ab9722) for 1 h at room temperature with HRP‐conjugated secondary antibodies (1:2000 goat anti‐rabbit, Abcam, ab6721; goat anti‐mouse, Abcam, ab6789). The blots were then incubated in stripping buffer (67.5 mM Tris, pH 6.8, 2% SDS, and 0.7% b‐mercaptoethanol) for 30 min at 50 uC and reprobed with a polyclonal rabbit anti‐β‐actin antibody (1:3000, mouse monoclonal, Abcam/ab6276) as the loading control. WB analysis was performed in triplicate. Differences were compared using *One‐way ANOVA*.

### 2.11 Immunohistochemistry

Rats were perfused with saline, followed by a 4% paraformaldehyde solution. Tissues were harvested and immersed in 25% sucrose solution (0.1 M PBS as solvent) until they sunk to the bottom of the tube. Each sample was cut into a 30 μm thick section (Microm International FSE, Thermo, USA). For observation of the coexpression of NLRP3, P2X7R, IL‐1β, and ionized calcium‐binding adapter molecule 1 (Iba1), the sections were incubated in primary antibodies anti‐Iba1 (1:200, goat multiple antibodies, Abcam, ab5076), anti‐NLRP3 (1:100, rabbit multiple antibodies, Abcam, ab214185), anti‐P2X7R (1:200, rabbit multiple antibodies, Abcam, APR‐004), and anti‐IL‐1β (1:50, rabbit multiple antibodies, Abcam, ab9722) at 4°C overnight. After washing with PBS, the sections were incubated in a solution containing Alexa Fluor® 488‐conjugated goat anti‐rabbit IgG H&G (1:200, Abcam, ab6717) and goat anti‐mouse IgG H&G (1:200, Abcam, ab97035) at RT for 2 h. The tissues were mounted in a mounting medium containing DAPI (Genepool/GPB18242). Brain sections were observed with a LEXT OLS4000 3D laser measurement microscope (Olympus), recorded with a digital camera, and processed in Adobe Photoshop.

### 2.12 Statistical analysis

The experimental data were analyzed using *SPSS 25.0* statistical software, and *GraphPad Prism 8.4.0* software was used for graphing. The Shapiro–Wilk test was used for assessing measurement data normality. The measurement data satisfied a normal distribution were expressed as the mean ± standard deviation $\left(\bar{X}\pm S\right)$ and were compared using *one‐way ANOVA* followed by post‐hoc Tukey pairwise comparison. For data not conforming to normality used the *Kruskal–Wallis* method with the rank sum test. Differences were considered statistically significant at *p* < 0.05.

## 3 RESULTS

### 3.1 Influences of depression phenotypes induced by different high‐fat diets on gut microbiota composition

Two kinds of different long‐term high‐fat diet consumption were carried out to induce depression phenotypes in ZDF rats. The ZDF + Purina group consumed a diet formulated with 32% fat, 13% protein, and 55% carbohydrates, while the ZDF + KK group consumed a diet formulated with 17.9% fat, 17.5% protein, and 48.5% carbohydrates. There was no difference in body weight (BW), FBG, FST, and OFT at week 0 (all *p* > 0.05). After sustained feeding of different HFD, BW in all ZDF groups decreased (*p* < 0.01), and among that in the ZDF + KK group decreased more significantly (Figure 1B). In terms of FBG, compared with the ZDF + Purina group, FBG values in the ZDF + KK group were significantly higher (*p* < 0.01) (Figure 1C). The score of FST in the ZDF + KK group increased, but there was no significant difference (*p* > 0.05); the number of horizontal and vertical spans decreased (both *p* < 0.01) (Figure 1D–F). When ZDF rats with depression modeling were performed using KK chow, the ZDF + KK group had lower mortality rates and appeared sooner in a state of polydipsia and depression.

**FIGURE 3:**
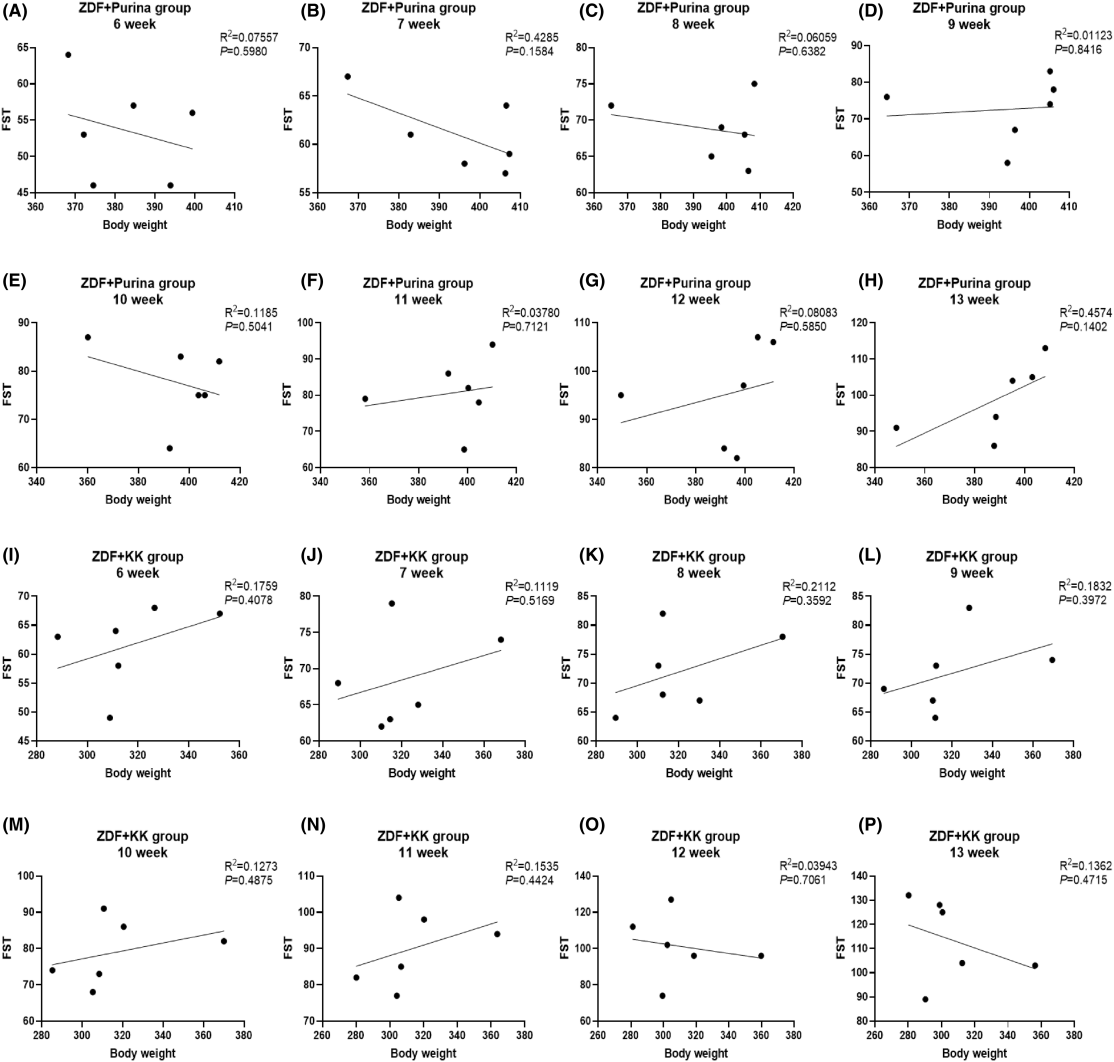
Correlation analyses between body weight and FST at weeks 6–13 for the ZDF + Purina group and ZDF + KK group.

Next, due to the statistically significant difference in the results of the FST from week 6 onwards, we performed correlation analyses between body weight and FST duration in weeks 6–13 for the ZDF + Purina group and the ZDF + KK group. The results showed that there was no correlation between body weight and FST in the ZDF + Purina and ZDF + KK groups (Figure 3).

**FIGURE 4:**
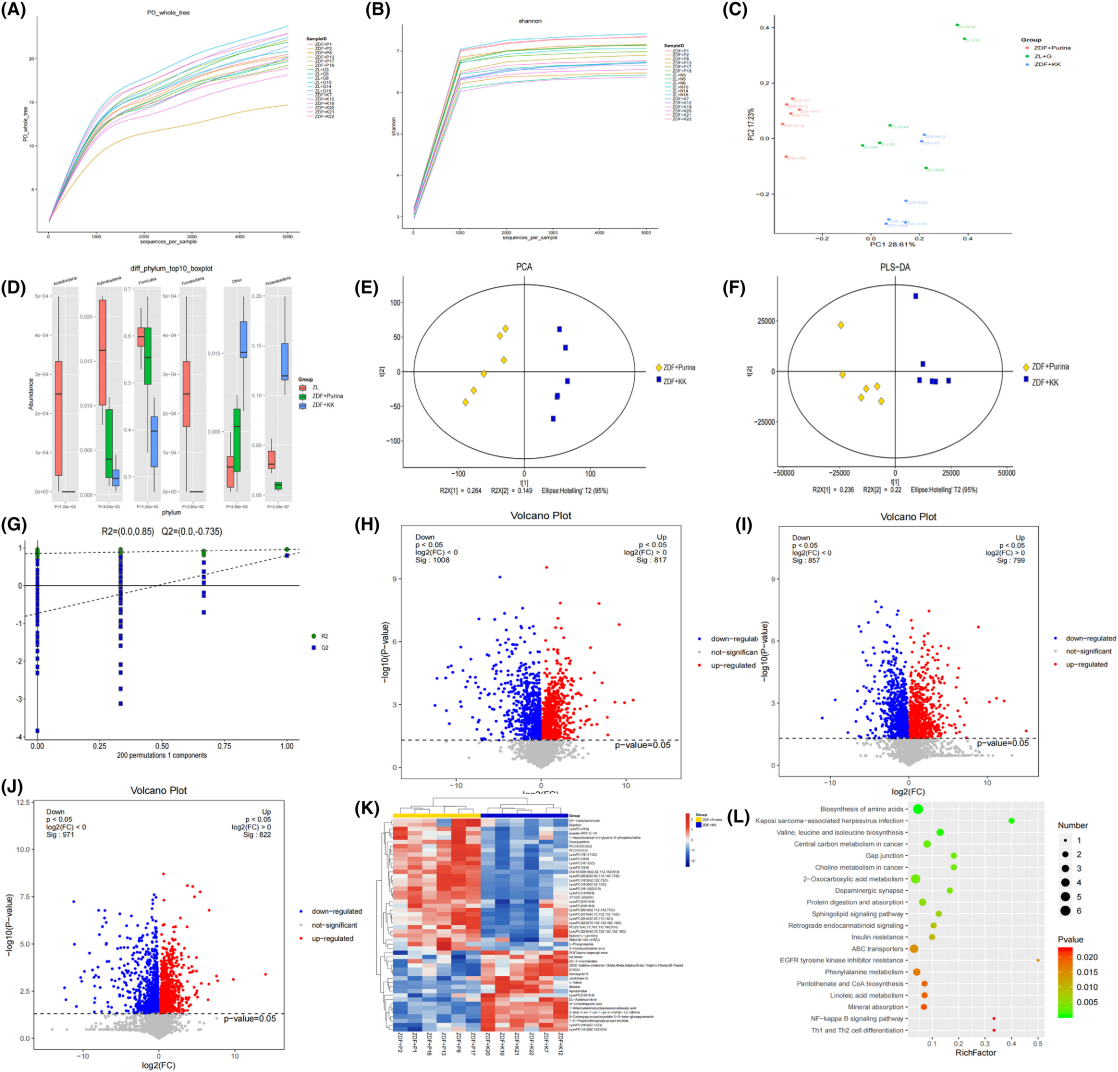
Differential metabolites and intestinal microbial population in different groups. (A, B) OTU rarefaction curve and Shannon index curve of samples; (C) PCA of rat intestinal flora; (D) Histogram of species structure analysis at the rat intestinal microflora level. Compared with the ZL group, the relative abundance of the phylum Proteobacteria in ZDF + Purina group increased by 12.48% (*p* < 0.01), and in the ZDF + KK group decreased by 10.03% (*p* < 0.01) compared with the ZDF + Purina group. The relative abundance of the Firmicutes phylum in the ZDF + Purina group decreased by 16.59% (*p* < 0.05) compared with that in the ZL group and in the ZDF + KK group increased by 21.93% (*p* < 0.01) compared with that in the ZDF + Purina group. The relative abundance of the Bacteroidetes phylum was increased by 4% (*p* < 0.05) in the ZDF + Purina group compared to the ZL group and decreased by 12.58% (*p* > 0.05) in the ZDF + KK group compared to ZDF + Purina group; (E) PCA analysis; (F) PLS‐DA analysis; (G) OPLS‐DA analysis; (H–J) Volcanic maps; (K) Hierarchical clustering results of significantly different metabolites; (L) Enrichment analysis diagram of differential metabolite KEGG pathways. *N* = 6 rats for each group.

We used alpha diversity analysis and observed that the rarefaction curve of OTU tends to be flat which indicated that the amount of sequencing data was reasonable and reflected the abundance of species indirectly; the Shannon dilution curve also tended to be flat, indicating that the amount of sequencing was sufficient (Figure 4A,B). Then, we used beta diversity analysis and observed that the samples in the ZL group were the most distant and well separated from the ZDF + Purina group using principal coordinates analysis (PCoA). It indicated that there was a significant difference in community structure between the two groups, i.e., the intestinal flora structure of ZDF rats was significantly changed when diabetic with depression. In contrast, the distribution of samples in the ZDF + KK group was gradually discrete compared with that in the Purina group, indicating that the effect of KK feed on the dysregulation of intestinal flora in diabetic rats with depression was weaker (Figure 4C).

16S rRNA sequencing was performed to evaluate the microbial diversity differences of depression phenotypes induced by different high‐fat diets. At the phylum level, we used *ANOVA* and observed that the intestinal flora of ZDF rats mainly consists of Proteobacteria, Firmicutes, Fusobacteria, Actinobacteria, Acidobacteria, etc., of which Proteobacteria and Firmicutes were the dominant groups of bacteria (Figure 4D).

### 3.2 Metabolome characteristics of depression phenotypes induced by different high‐fat diets

PCA score plot showed that the QC samples were tightly clustered in both positive and negative ion modes, demonstrating their good reproducibility and stability (Figure 4E). As shown in Figure 4F,G, the partial least squares‐discriminant analysis (PLS‐DA) model significantly differentiated the ZDF + Purina group from all the other groups, indicating that the occurrence of diabetes caused significant changes in the concentration of intestinal metabolites. The combined application of fold change (FC) analysis and *t*‐test to analyze the significance of metabolite changes between the two samples resulted in a volcano plot. The red points in the volcano plot are metabolites with FC > 1.5 and FDR‐*p* < 0.05, which are the differential metabolites between the two groups (Figure 4H–J). And, there were significant upregulated/downregulated differential metabolites between the three groups. Further hierarchical clustering revealed 235 significantly different metabolites between the ZL and ZDF + Purina groups; and 185 significantly different metabolites between ZDF + Purina and ZDF + KK groups, including rising bile acids [glycocholic acid, deoxycholic acid, cholic acid, etc.], significantly declining fatty acids [linoleic acid, oleic acid, trans‐vaccenic acid, etc.], some purines, and their derivatives [Xanthine, Hypoxanthine, N6‐methyladenine] (Figure 4K). It is suggested that the Purina#5008 diet caused significant changes in plasma metabolites in diabetic depression rats to cause disorders of glucolipid metabolism. As shown in Figure 4L, enrichment analysis of KEGG pathway showed that the metabolic pathways with the most significant effects between the ZDF + Purina and ZDF + KK groups were Th1 and Th2 cell differentiation, EGFR tyrosine kinase inhibitor resistance, Pantothenate and CoA biosynthesis, Linoleic acid metabolism, and Phenylalanine metabolism.

### 3.3 taVNS alleviated depressive behaviors in ZDF rats

Before modeling, there was no difference in the duration of immobility time in the FST and score of horizontal and vertical movement of OFT between the groups (all *p* > 0.05). After feeding with the high‐fat diet Purina #5008 for 8 weeks, significantly depressive‐like behaviors were observed in all the ZDF groups (all *p* < 0.01). TaVNS intervention started at week 8 for the next 6 weeks. The results showed that immobility time in the ZDF + taVNS group at weeks 10–14 was significantly shorter than that in the ZDF group (*p* < 0.01), while it tended to decrease in ZDF + sham‐taVNS group. However, it was significantly higher than that of ZDF + taVNS group at weeks 12–14 (*p* < 0.01) (Figure 2D). Additionally, at weeks 8–14, the horizontal movement scores of the ZDF group were significantly lower than those in the ZL group (*p* < 0.01); after taVNS intervention, at week 14, the scores of horizontal movement in the ZDF + taVNS group were significantly higher than that of the ZDF group (*p* < 0.01) and ZDF + sham‐taVNS group (*p* < 0.01). It is the same as the score of vertical movement (Figure 2E,F).

### 3.4 taVNS improved glucose metabolism disorders in the ZDF rats

There was no difference in FBG and BW groups (all *P* > 0.05) at baseline. During the modeling, these two indicators in the ZDF groups were consistently higher than those in ZL group (*p* < 0.01). After taVNS intervention, compared with the ZDF group, FBG decreased at 10 w to 14 w in the ZDF + taVNS group (*p* < 0.01), but there was no significant difference in the ZDF + sham‐taVNS group (*p* > 0.05). The BW values of all ZDF rats, after feeding a high‐fat diet (Purina #5008), were always higher than those in the ZL group (*p* < 0.01). After taVNS interventions, from the 9th week onward, the BW of the ZDF group tended to decrease but was still higher than that in the ZL group (*p* < 0.01). BW values of rats in the ZDF + taVNS group were much higher than those in the ZDF group, but there was no significant difference in the ZDF + sham‐taVNS group (*p* > 0.05) (Figure 2C,G).

Compared with ZL group, rats in the ZDF group showed a high level of HbA1c (*p* < 0.01) and glucagon (*p* < 0.01), and a low level of insulin (*p* < 0.01). Interestingly, taVNS reversed these trends compared with the ZDF group. However, the concentration of these indicators showed no significant difference in the ZDF + sham‐taVNS group (Figure 2H–J).

### The expression of P2X7R, NLRP3, and IL‐1β in PFC

**FIGURE 5:**
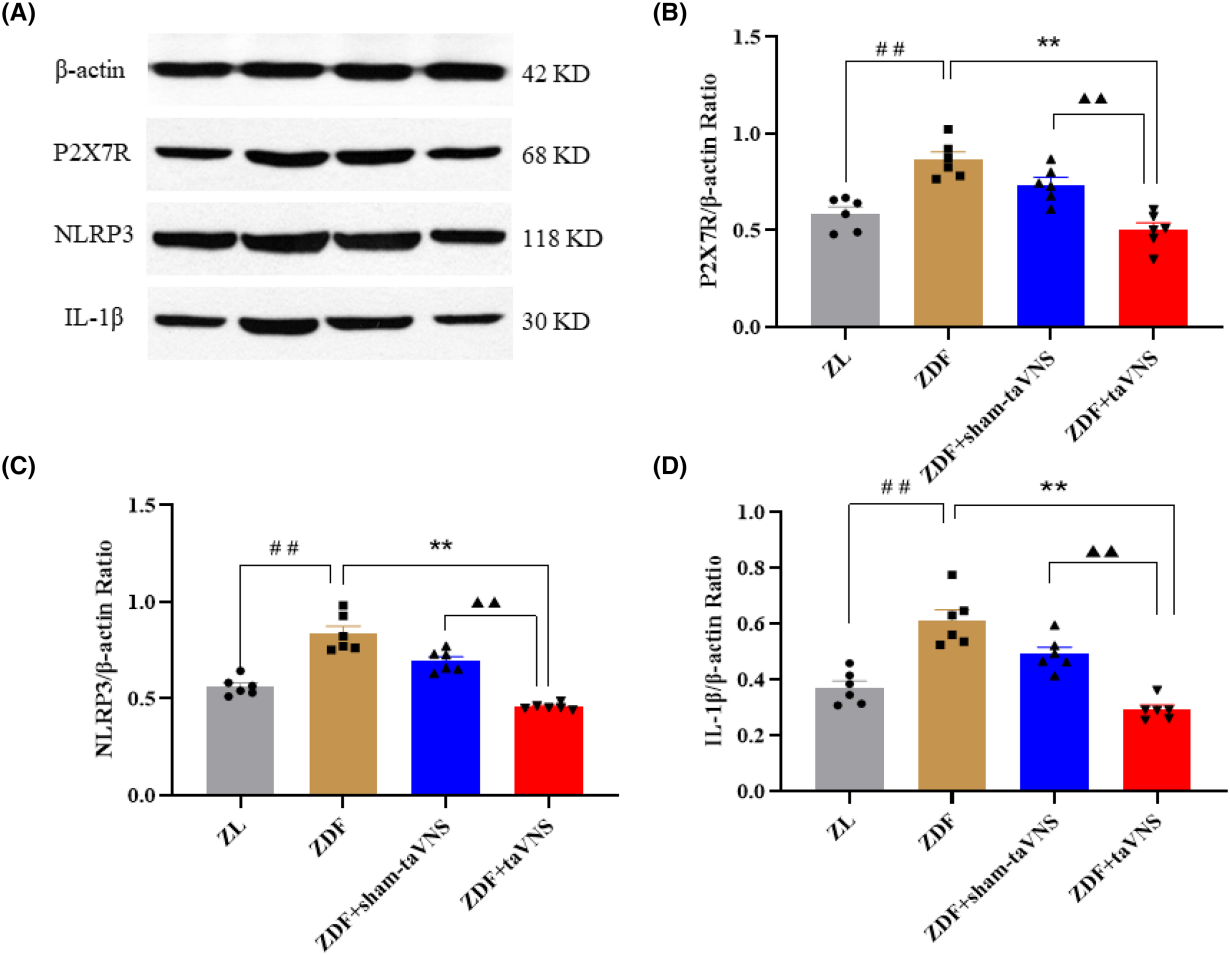
The effect of taVNS on the expression of P2X7R, NLRP3, and IL‐1β in the PFC. The values are presented as the mean ± SD. *One‐way ANOVA* with *Tukey's* post hoc *test* was performed for statistical analysis. *N* = 6 rats for each group. ^##^ *p* < 0.01 vs. ZL group, **p < 0.01 vs. ZDF group, ^▲▲^ *p* < 0.01 vs. ZDF + taVNS group.

We observed that the expression of P2X7R, NLRP3, and IL‐1β was higher in the PFC of ZDF group compared to that in the ZL group (*p* < 0.01). In comparison with ZDF group, the lower expression level of these three indicators was shown in the ZDF + taVNS group (*p* < 0.01), and no significant difference was observed in the ZDF + sham‐taVNS group (*p* > 0.05). The expression levels were much higher in the ZDF + sham‐taVNS group than that in the ZDF + taVNS group (*p* < 0.01) (Figure 5).

**FIGURE 6:**
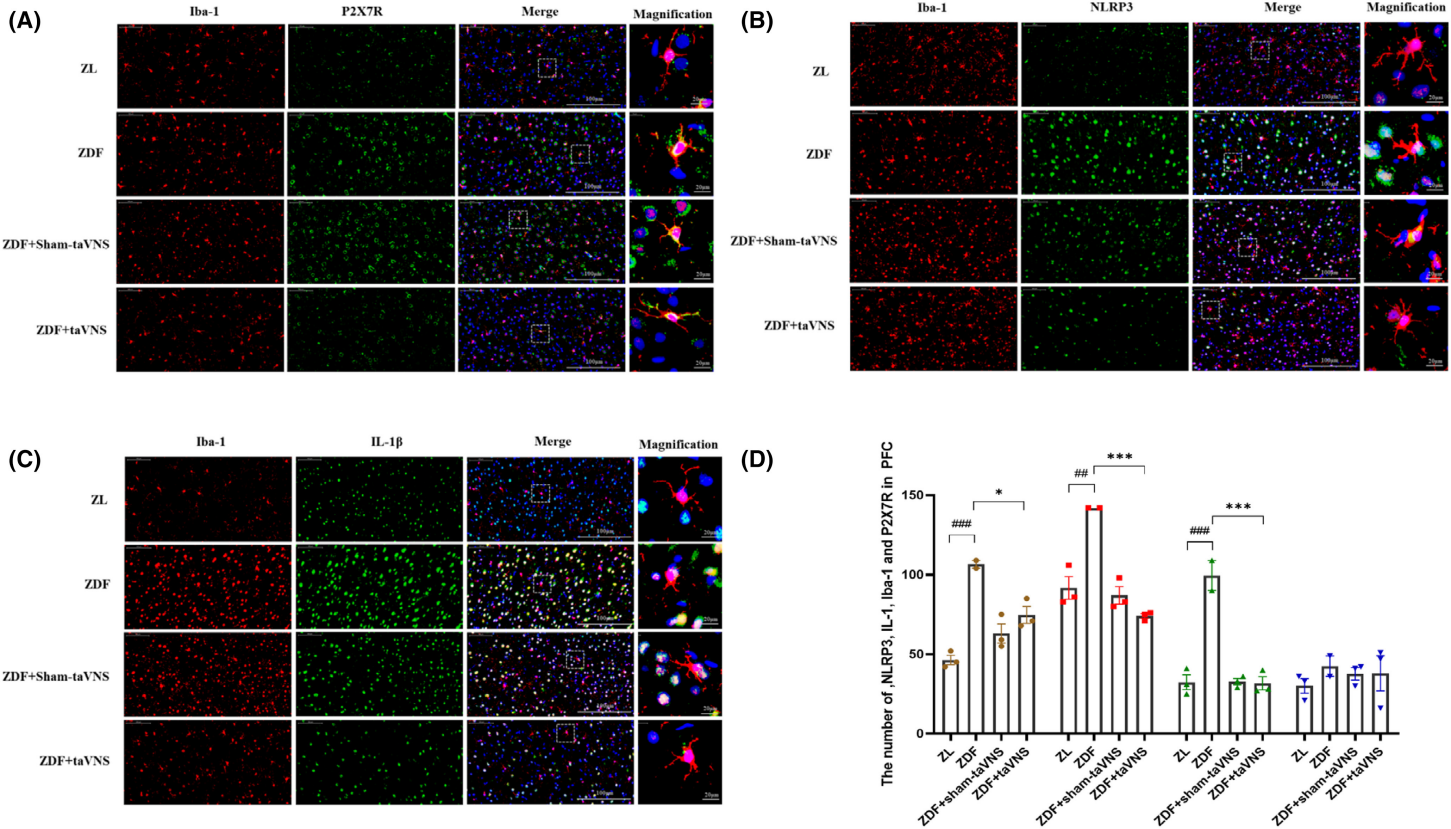
The effect of taVNS on the expression of P2X7R, NLRP3, and IL‐1β and the number of activated or resting state microglia in the PFC. (A–C) Immunofluorescence staining of PFC sections in each group of rats (P2X7R, NLRP3, and IL‐1β). Compared with the ZL group and ZDF + taVNS group, representative confocal images of the PFC show increased expression of P2X7R, NLRP3, and IL‐1β (green) in the ZDF group and ZDF + sham+taVNS group. The microglia transformed from a ramified morphology (ZL group) to an ameboid form (ZDF group) to a ramified state (ZDF + taVNS group) and the ZDF + sham+taVNS group showed no obvious change compared with the ZDF group. Compared with the ZL group and ZDF + taVNS group, the coexpression between P2X7R, NLRP3, IL‐1β (green), and microglia (red) increased in ZDF group, while the result was reversed in the ZDF + taVNS group, but not in the ZDF + sham + taVNS group. P2X7R (green), Iba‐1 (red), DAPI (blue), Merge (yellow), 40× objective lens, scale bar: 100 μm and 20 μm magnification. The values are presented as the mean ± SD. *One‐way ANOVA* with *Tukey's* post hoc *test* was performed for statistical analysis. ^##^ *p* < 0.01 vs. ZL group, **p < 0.01 vs. ZDF group, ^▲▲^ *p* < 0.01 vs. ZDF + taVNS group. (D) Number of activated or resting state microglia in the PFC. *N* = 3 rats for each group. ^#^ *p* < 0.05, ^##^ *p* < 0.01, ^###^ *p* < 0.001 vs. ZL group, *p < 0.05, ***p < 0.001 vs. ZDF group.

Immunofluorescence staining of Iba‐1 in microglia was used to observe the morphology of microglia in the PFC of each group to determine the resting or activated state of microglia. The results showed that the microglia in the ZL group were fewer and in a resting state, mainly showing smaller cytosol and elongated branches, while they were more abundant and in an activated state in the ZDF group, which mainly showed shorter and thicker cytosolic components and irregular enlargement of the cell body compared with the ZL group. The morphology of microglia in the taVNS group was not activated compared with that in the ZDF group. P2X7R was mainly expressed on the microglial membrane and the co‐expression of P2X7R in the ZL and ZDF + taVNS groups was lower than that in the ZDF group (*p* < 0.01). Similarly, the expression of NLRP3 and IL‐1β in the ZDF + taVNS group was significantly decreased compared with that in the ZDF group (*p* < 0.01) (Figure 6).

Additionally, there was a decrease in the number of activated or resting state microglia in PFC region after taVNS, with a significant decrease in the number of NLRP3, IL‐1β, and Iba‐1 (*p* < 0.05, *p* < 0.001, *p* < 0.001, respectively), and a decrease in the number of P2X7R but no significant difference (*p* > 0.05) (Figure 6).

## 4 DISCUSSION

### 4.1 TaVNS alleviates depression‐like phenotype induced by a high‐fat diet in ZDF rats

Many studies have suggested that a high‐fat diet is a risk factor for the development of induced depression.^34^ Long‐term high‐fat diets promote chronic inflammatory responses in areas such as the cerebral cortex, hippocampus, and even by modulating gut microbes to influence the body's emotional state,^35^ making the neuroinflammatory response to diabetic with depression. Evidence suggests that a high‐fat diet combined with streptozocin intraperitoneal injection increases the amount of intestinal microflora mimicry in diabetic rats, increases the ratio of Clostridium/thick‐walled phylum, increases the level of colonic inflammatory markers, and overactivated neuroglia to promote neuroinflammatory responses, which in turn induces depressive‐like phenotype in diabetic rats.^36^

The ZDF rats, in our experiment, fed high‐fat chow for 0–8 weeks showed a continuous increase in FBG, an increase in FST immobility time, and a decrease in scores of OFT. In particular, the depressive behaviors induced by the high‐fat Purina #5008 diet showed a potential mechanism relevant to inflammatory markers. At the end of the intervention, taVNS reversed the high level of FBG and obviously depressive‐like behaviors in ZDF group, but sham‐taVNS have no similar effects. Additionally, the concentrations of plasma glucose‐related biochemical indexes, HbA1c, and glucagon in the ZDF + taVNS group were significantly lower than those in the ZDF group and ZDF + sham‐taVNS group. In comparison, insulin concentrations were significantly higher than those in the ZDF group.

### 4.2 TaVNS reverses the activated state of microglia in PFC of depressive‐like behaviors in ZDF rats

When taVNS stimulates the auricular branch of vagus nerve (ABVN), the brain receives information from the vagus afferent fibers. The vagus nerve enters the central nervous system in the nucleus tractus solitarius (NTS).^37^ From the NTS, there are direct afferent projections to the parabrachial complex (PB); from here, projections are sent to the locus coeruleus (LC) and raphe nuclei.^38^ It is worth noting that in previous studies, researchers used iontophoretic injections of biotinylated dextran amine (BDA) or Phaseolus vulgaris leucoagglutinin (PHA‐L) in the NTS combined with immunocytochemical labeling of tyrosine hydroxylase (TH) to find that NTS efferent synapse directly with noradrenergic neurons in the LC.^39^ Further ascending projections from the PB ascend to higher brain regions like the amygdala.^40^ Anatomical evidence^41^ suggests that most of the projection fibers of PFC neurons are located in the basolateral amygdala (BLA). There are many excitatory projection neurons (PNs) in the BLA, which are structurally and functionally connected to other brain regions, and different BLA PNs have specific target brain regions and are involved in different physiological activities.^42^, ^43^ The presence of projection neuron synapses receiving unidirectional inputs from the dmPFC in the BLA PNs was found in viral reverse tracer experiments.^44^

Physiologically, microglia contribute to CNS synaptogenesis and maintenance of brain homeostasis and can also be hyperactivated in a variety of contexts, such as inflammation, infection, trauma, and apoptosis, and activated microglia secrete inflammatory cytokines into the extracellular environment, participating in the pathogenesis of neuroinflammation and mood disorders.^23^ Reduced medial PFC volume is one of the best‐reported neurological abnormalities in depression. It is also strongly associated with markers of depression progression, including an increased number of depression episodes, prolonged disease duration, and treatment resistance.^45^ Preclinical studies found that chronic unpredictable mild stress (CUMS) induced activation of microglia in the PFC,^46^ with a significantly increasing expression of Iba‐1, and caused obviously changes in depression‐like behaviors. Additionally, the up‐expression of NLRP3, caspase‐1, ASC, and IL‐1β and the reversal of microglia‐associated proinflammatory imbalance relieved the depressive‐like behaviors in the PFC. Our previous fMRI results showed that taVNS significantly activates the dorsolateral PFC by stimulating the auricular branch of the vagus nerve (ABVN), causing a blood oxygenation level‐dependent signal response in the dorsolateral PFC.^47^

The results showed that the microglia in PFC of ZDF group were more numerous and in the activated state, mainly manifested by shorter and thicker cytosolic branches than those in the ZL and ZDF + sham‐taVNS groups. Compared with the ZDF group, taVNS changed the microglia from the activated to resting state, and they were also more activated than those in the ZDF + sham‐taVNS group. Therefore, we suggest that high‐fat diet‐induced excessive microglial activation in ZDF rats and a central inflammatory response, while taVNS, but not sham‐taVNS, reversed this activated state of microglia.

### 4.3 Involvement of P2X7R/NLRP3/IL‐1β signaling pathway in PFC on the antidepressant effect of taVNS in ZDF rats

Microglia‐specific activity results from a combination of environmental stimulation and cellular states. The purinergic system is one of the fundamental signaling systems that establish microglial behavior under broad‐spectrum conditions.^48^ The P2X7 receptor is a trimeric complex with variable conformational switching between open and closed states, and participates in the immune response by activating immune cells to release inflammatory factors. P2X7 receptors are predominantly expressed in microglia, and microglial activation is dependent on P2X7 receptors.^49^ Abnormalities in brain purinergic transmission are part of the pathogenesis of neuropsychiatric disorders such as depression.^50^ ATP, as a neurotransmitter, belongs to the purinergic signaling system and can regulate nervous system function. Adverse stress signals stimulate nerve endings to excessively release glutamate, which not only produces excitotoxicity but also causes elevated extracellular ATP levels in the brain, leading to a cascade response that promotes activation of microglia P2X7 receptors and elevated IL‐1β levels, inducing inflammation in the CNS and ultimately leading to depression.^51^ In addition to depression, abnormal P2X7 receptor function also impacts metabolic disorders.^52^

NLRP3 is an important pattern recognition receptor in the cytoplasm. Oligomerized NLRP3 recruits ASCs through the homotypic PYD‐PYD structural domain and induces ASCs to aggregate into macromolecular foci called ASC spots.^53^ ASC spots subsequently recruit procaspase‐1 through the homotypic CARD‐CARD structural domain to form the NLRP3‐ASC‐caspase‐1 protein complex, the NLRP3 inflammasome. An activated NLRP3 inflammasome is important in maintaining homeostasis and defending against pathogen invasion. However, an overactivated NLRP3 inflammasome induces procaspase‐1 self‐cleavage and activation, causing maturation of pro‐inflammatory cytokines and release of inflammatory factors, causing central nervous system disorders, and promoting the progression of inflammatory diseases such as depression.^54^ When NLRP3 inflammasomes are overactivated, immune cells secrete excessive inflammatory factors (e.g., IL‐1β), all of which are closely associated with insulin resistance, metabolic syndrome, and diabetic progression.^55^ Studies have shown that NLRP3 inflammasome deletion significantly reduces diabetes‐induced depressive phenotypes.^56^ Upstream P2X7 receptors can activate the NLRP3 inflammasome to generate an inflammatory cascade response, causing the release of the proinflammatory cytokine IL‐1β. This signaling pathway may be associated with the pathogenesis of diabetes‐associated depression which would be a key target for treating diabetes‐associated depression.^17^, ^57^

Our previous study found that taVNS reduced the severity of depression in patients and that the mechanism of efficacy was associated with increased functional connectivity between the rostral ACC and the mPFC.^20^ Therefore, we suggest that taVNS modulates the central inflammatory response to treat mental disorders by stimulating the ABVN through the vagal trunk and projecting it through the brainstem to the PFC. In this study, the depressive‐like behaviors and increased expression of P2X7R, NLRP3, and IL‐1β in the PFC were observed in the ZDF group induced by a high‐fat diet. After continuous long‐term taVNS intervention, the levels of P2X7R, NLRP3, and IL‐1β were significantly reduced, and depressive‐like behavior was alleviated in the ZDF + taVNS group. These results suggested that the P2X7R/NLRP3/IL‐1β signaling pathway in the PFC might be one of the central antidepressant mechanisms by which taVNS alleviates depression‐like behaviors in ZDF rats.

## 5 LIMITATIONS AND FUTURE PROSPECTS

This study has some limitations. In assessing the choice of behavioral experiments, we took into account the fact that the animals showed depressive‐like behavior accompanied by elevated blood glucose, so we did not use sucrose preference tests, and record rats' diving time in the forced swimming test and total distance traveled in the open field to reduce the rat death.

As the focus of this experiment explored the central anti‐inflammatory mechanism of action in the prefrontal cortex, in future experiments we will further elucidate the central‐peripheral connection by detecting inflammatory factors in peripheral blood. Due to the fact that this animal model has a wound that is prone to infection, which makes it impossible to place the antagonist delivery device on the animal's head for a long period, and that it is difficult to carry out the receptor knockout technique on the basis of this co‐morbid animal model, we need to think about how to test more indexes in the next step of the experiments.

## 6 CONCLUSION

TaVNS can alleviate depression‐like behaviors induced by a high‐fat diet in ZDF rats which might be due to anti‐inflammation by up‐regulation of the P2X7R/NLRP3/IL‐1β signaling pathway in PFC. It will provide a new idea for exploring techniques in the treatment of metabolic disorders with depression from the perspective of anti‐inflammation.

## AUTHOR CONTRIBUTIONS

LS: Conceptualization, Project administration, Formal analysis, Writing – original draft, Writing – review & editing, and Funding acquisition. ZY: Data section, Writing – original draft, Writing – review & editing. WY: Conceptualization, Project administration, and Writing. ZZ: Project administration. XC: Project administration and Writing. WY: Project administration. RP: Conceptualization, review & editing, and funding acquisition.

## FUNDING INFORMATION

The work is supported by the National Natural Science Foundation of China (82174519; 82004181; GZ1236), lnnovation Project of China Academy of Chinese Medical Sciences (CI2021A03405; ZZ15‐YQ‐048), Fundamental Research Funds for the Central Public Welfare Research Institutes (ZZ202219002, ZZ‐YQ2023006), and Young Elite Scientists Sponsorship Program by CAST (2021‐2023ZGZJXH‐QNRC002).

## CONFLICT OF INTEREST STATEMENT

The authors declare that they have no conflicts of interest.

## Supporting information


File S1.

## Data Availability

Data available on request from the authors.
