# Supplementary material for: Transcutaneous vagus nerve stimulation modulates depression‐like phenotype induced by high‐fat diet via P2X7R/NLRP3/IL‐1β in the prefrontal cortex
Source: CNS Neurosci Ther. 2024 May 16;30(5):e14755. doi: 10.1111/cns.14755 (PMC11097256; doi:10.1111/cns.14755)

Full unedited blot for Figure 5

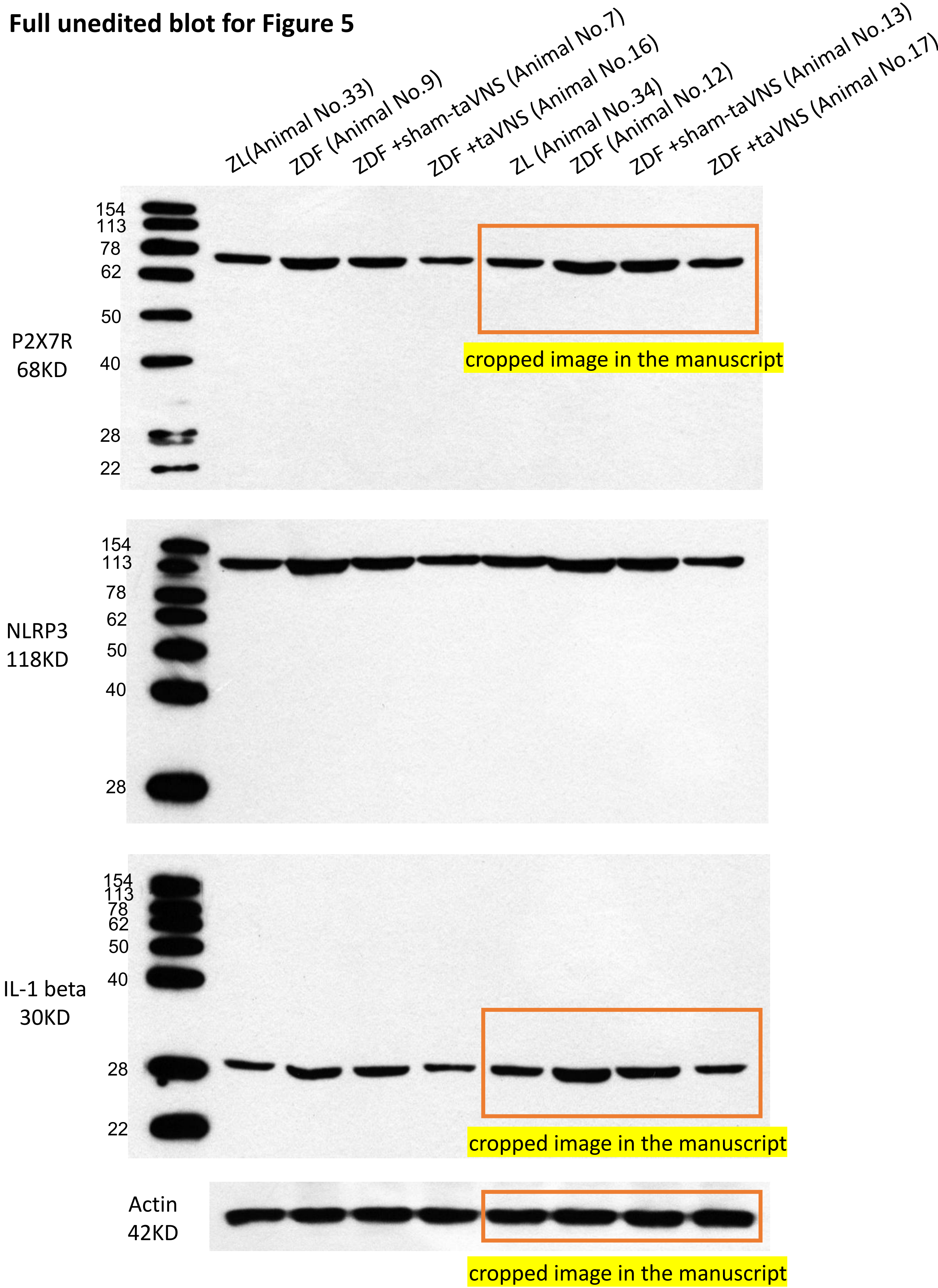

Full unedited blot for Figure 5

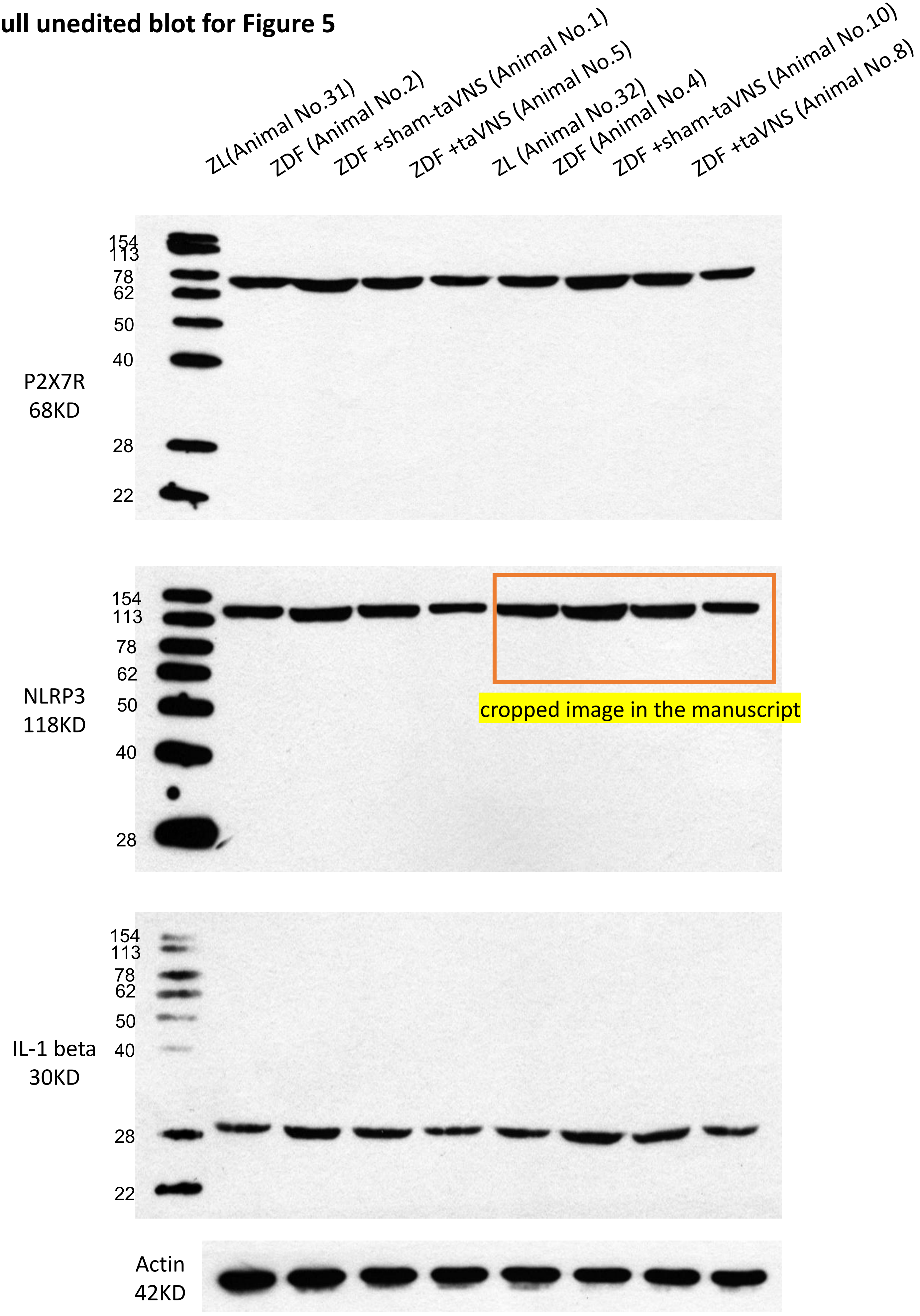

Full unedited blot for Figure 5

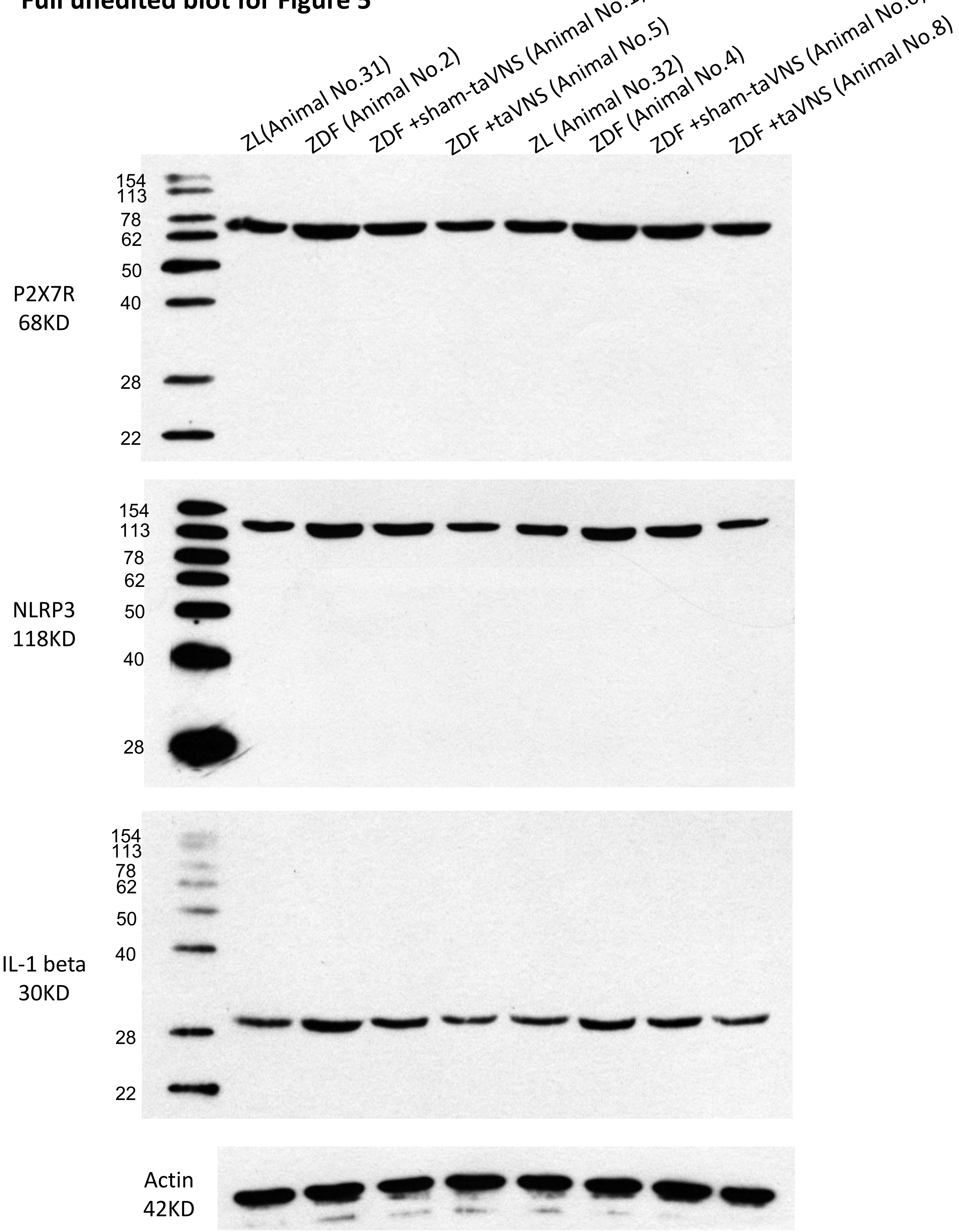

Supplement: Supplementary file 1 — File S1. [file CNS-30-e14755-s001.zip › WB-Supplemental Files.pdf]
